# Evolution of orthologous tandemly arrayed gene clusters

**DOI:** 10.1186/1471-2105-12-S9-S2

**Published:** 2011-10-05

**Authors:** Olivier Tremblay Savard, Denis Bertrand, Nadia El-Mabrouk

**Affiliations:** 1 Department of Computer Science (DIRO), University of Montreal, Montreal, Quebec, Canada; 2 Computational and Mathematical Biology, Genome Institute of Singapore, Singapore

## Abstract

**Background:**

Tandemly Arrayed Gene (TAG) clusters are groups of paralogous genes that are found adjacent on a chromosome. TAGs represent an important repertoire of genes in eukaryotes. In addition to tandem duplication events, TAG clusters are affected during their evolution by other mechanisms, such as inversion and deletion events, that affect the order and orientation of genes. The DILTAG algorithm developed in [1] makes it possible to infer a set of optimal evolutionary histories explaining the evolution of a single TAG cluster, from an ancestral single gene, through tandem duplications (simple or multiple, direct or inverted), deletions and inversion events.

**Results:**

We present a general methodology, which is an extension of DILTAG, for the study of the evolutionary history of a set of orthologous TAG clusters in multiple species. In addition to the speciation events reflected by the phylogenetic tree of the considered species, the evolutionary events that are taken into account are simple or multiple tandem duplications, direct or inverted, simple or multiple deletions, and inversions. We analysed the performance of our algorithm on simulated data sets and we applied it to the protocadherin gene clusters of human, chimpanzee, mouse and rat.

**Conclusions:**

Our results obtained on simulated data sets showed a good performance in inferring the total number and size distribution of duplication events. A limitation of the algorithm is however in dealing with multiple gene deletions, as the algorithm is highly exponential in this case, and becomes quickly intractable.

## Background

Gene duplication is a fundamental process in the evolution of species [2], especially in eukaryotes [3-8], where it is believed to play a leading role for the creation of novel gene functions. Several mechanisms are at the origin of gene duplications, among them tandem repeat through unequal crossing-over during recombination. As this phenomenon is facilitated by the presence of repetitive sequences, a single duplication can induce a chain reaction leading to further duplications, eventually creating large *Tandemly Arrayed Gene* (*TAG*) *clusters:* groups of paralogous genes that are adjacent on a chromosome. TAGs account for about one-third of the duplicated genes in eukaryotes [9]. In human, they represent about 15% of all genes [10]. In *Arabidopsis*, 17% of the total predicted genes are members of TAG clusters [11], and in maize, about 35% of the genes were predicted to belong to TAG clusters [12].

Deciphering the evolutionary history of a TAG cluster is important to provide new insights into the mechanisms of gene amplification, and to answer several questions regarding the nature and size of duplication and other evolutionary events that have shaped TAG clusters. In most biology-oriented studies, a gene tree is obtained by applying a classical phylogenetic method to an alignment of the amino acid sequences corresponding to the collected gene sequences, and a duplication scenario is proposed for the gene family, based on a careful analysis of this gene tree (see for example [9] for the study of the 22-kDA prolamin gene amplification in grass genomes). Although such manual analysis may be useful to propose amplification scenarios for families of limited size and simple organization, it is usually impractical to infer more general evolutionary scenarios for large TAG clusters affected, in addition to duplications, by other events such as segmental deletion, that may lead to gene loss, and rearrangements (such as inversions or inverted duplications), that may affect gene order and transcriptional orientations.

The *tandem-duplication model of evolution*, first introduced by Fitch in 1977 [13], assumes that, from a single ancestral gene at a given position in the chromosome, the locus grows through a series of consecutive duplications placing the newly created copy next to the original one. Such tandem duplications may be *simple* (duplication of a single gene) or *multiple* (simultaneous duplication of neighbouring genes). Based on this idea, a number of theoretical studies have considered the problem of reconstructing the tandem-duplication history of a TAG cluster [14-17]. However, due to rearrangements and losses, it is often impossible to reconstruct a duplication history for a TAG cluster [18], even from well-supported gene trees. In [19], we considered a generalization of the tandem-duplication model allowing for inversions. The model was then extended in [20] to the study of orthologous TAG clusters in different species. A similar work, considering more operations (translocations, fusions, fissions, duplications in tandem or not), but requiring more preliminary information (gene and species trees with branch length) has also been done [21]. Various other heuristic and probabilistic methods have been developed for reconstructing a hypothetical ancestral sequence and a most parsimonious set of duplications (in tandem or not) and other evolutionary events leading to the observed gene cluster [22-25]. They are based on a preprocessing of a self-alignment dot-plot of a cluster, or the dot-plot of a pairwise-alignment of two clusters. Although these methods are useful to infer evolutionary events in well-conserved regions, they are less appropriate when there is a lot of noise in the dot-plots due to the alignments of nunfunctional regions which are continuously affected by mutations. In both of our previous cited methods [19,20], only simple duplications were considered. This assumption, while allowing for exact algorithmic solutions, is an important limitation to its applicability (see for example [26]). For this reason, we have developed a more general heuristic, the DILTAG algorithm [1], allowing us to infer a set of optimal evolutionary histories for a gene cluster in a single species, according to a general cost model involving variable length duplications, in tandem or inverted, deletions and inversions. Experiments on simulated data showed that the most recent evolutionary events can be inferred accurately when the exact gene trees are used. Despite the uncertainty associated with the deeper parts of the reconstructed histories, they can be used to infer the duplication size distribution with some precision. DILTAG has been used recently in [27] to infer an evolutionary scenario for the Maltase gene clusters in *Drosophila.*

A clear limitation of DILTAG is the fact that it is applicable only to a single cluster. The benefit of an extension to multiple species is obvious, as comparative genomics is clearly a more appropriate approach to infer loss and inversion events. In particular, considering an outgroup may help in choosing among many possible optimal evolutionary scenarios for a gene cluster.

In this paper we present an extension of DILTAG to the study of a set of orthologous TAG clusters in multiple species. In other words, in addition to multiple duplication (in tandem or inverted), deletion and inversion events, the speciation events reflected by a given phylogenetic tree for the set of species are also taken into account. We develop Multi-DILTAG, a heuristic algorithm that is shown on simulated data sets to be very accurate in inferring the total number and size distribution of duplication events.

## Methods

### Data

Preliminary to all the developments in this paper is the identification of *m* orthologous TAG clusters in *m* genomes of interest. In other words, given a gene family *F* of interest, a tandemly arrayed sequence (called TAG cluster) of paralogous genes from *F* has already been identified in each genome, and such *m* TAG clusters have already been pointed out as orthologs. For example, gene orders and clusters orthology for the protocadherin gene family has been identified for human and several other mammalian and fish species [28,29].

**Figure 1:**
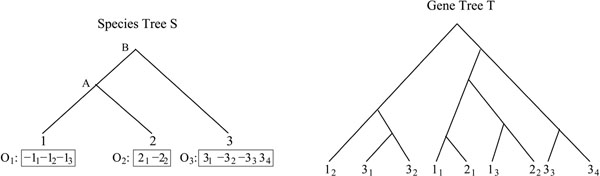
**Species and gene trees for the three genomes 1, 2, 3.** Left: The species tree for three genomes 1, 2, 3. Three orthologous TAG clusters {*O*_1_, *O*_2_, *O*_3_} are identified in the three genomes. The notation *j_i_* denotes the *i*th gene in genome *j.* Right: A gene tree for the gene family.

We denote by  the set of *m* TAG clusters, *i.e.* for 1 ≤ *i* ≤ *m*, *O_i_* is the signed order of the family members in genome *i.* The sign (+/–) of a gene represents its transcriptional orientation. In addition to the observed gene orders, we also assume that a gene tree is available for the *TAG family*, *i.e.* the set of genes contained in the *m* TAG clusters. A *gene tree T* for a TAG family is a rooted binary tree with labelled leaves, where each label represents an unsigned gene copy. A leaf labelled by a gene copy in genome *i* is said to *belong to genome i.* For conciseness, we make no distinction between a leaf and its label. The pair  is called the *ordered gene tree* for the gene family. Finally, we assume that the species tree, reflecting the speciation history of the *m* considered genomes, is also available. See Figure 1 for an example.

### The evolutionary model

Our evolutionary model is an extension of the one introduced by Fitch [13] for TAGs, which considers only tandem duplications resulting from unequal crossing-over during meiosis. However, TAGs are shaped during their evolution by other events affecting the gene order, orientation and content of the clusters. For example, Shoja and Zhang [10] have observed that more than 25% of all neighbouring pairs of TAGs in human, mouse and rat have non-parallel orientations. The Fitch model of evolution does not apply to such data. Our model extends the Fitch model of evolution by considering deletion events affecting gene content, as well as inversion and inverted duplication events affecting gene orientation. Below is a formal definition of the evolutionary model considered in this paper. In this definition, a *cherry* of *T* is a pair of leaves (*l*, *r*) separated by a single vertex, called its *root.*

**Definition 1:** An *evolutionary history* for  is a sequence of ordered gene trees , such that for each  is a set of *n_k_* gene orders corresponding to orthologous TAG clusters on *n_k_* genomes, where:

1. *T*^1^ is a tree consisting of a single leaf *u*, and .

2. For 1 ≤ *k* <*h*, there is a unique genome *i* such that  can be obtained from  by applying one of the following evolutionary events on :

(a) **Duplication:** A sub-sequence (*u_p_*, *u_p_*_+1_, …, *u_q_*) of  is replaced by a sequence of new elements (*l_p_*, *l_p_*_+1_, …, *l_q_*, *r_p_*, *r_p_*_+1_, …, *r_q_*), where, for each *p* ≤ *x* ≤ *q*, *l_x_* and *r_x_* have the same sign as *u_x_*. Moreover, each leaf *u_x_* in *T^k^* is replaced by the cherry (*l_x_*, *r_x_*)*.*

(b) **Inverted-duplication:** A sub-sequence (*u_p_*, *u_p_*_+1_, …, *u_q_*) of  is replaced by (*–*(*l_q_*), *–*(*l_q–_*_1_), …, *–*(*l_p_*), *r_p_*, *r_p_*_+1_, …, *r_q_*) or (*l_p_*, *l_p_*_+1_, …, *l_q_*, *–*(*r_q_*), *–*(*r_q_*_–1_), …, *–*(*r_p_*)), where, for each *p* ≤ *x* ≤ *q*, *l_x_* and *r_x_* have the same sign as *u_x_.* Moreover, each leaf *u_x_* of *T_k_* is replaced by the cherry (*l_x_*, *r_x_*)*.*

(c) **Inversion:** A sub-sequence (*u_p_*, *u_p_*_+1_, *…*, *u_q_*) of  is replaced by (*–*(*u_q_*), *–*(*u_q_*_–1_), …, –(*u_p_*)) and *T^k^* remains unchanged.

(d) **Deletion:** A sub-sequence (*u_p_*, *u_p_*_+1_, *…*, *u_q_*) of  is deleted, and the corresponding leaves (genes) are removed from *T^k^* (each removed gene corresponds to a gene loss).

(e) **Speciation:** The complete order  is replaced by {(*l*_1_, …, *l_t_*), (*r*_1_, …, *r_t_*)}, where, for each *1* ≤ *x* ≤ *t*, *l_x_* and *r_x_* have the same sign as *u_x_.* Moreover, each leaf *u_x_* belonging to genome *i* is replaced by the cherry (*l_x_*, *r_x_*)*.*

Any evolutionary history  for  induces a unique species tree *S* obtained from the speciation events of . We say that  is *consistent with S.*

**Figure 2:**
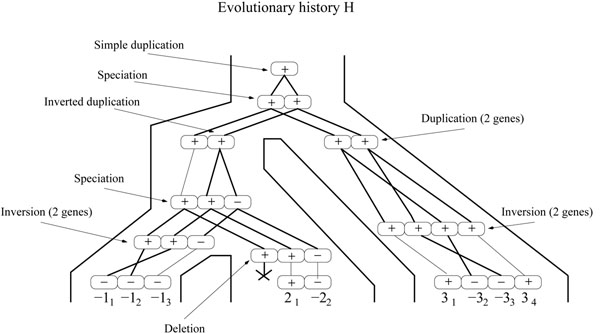
**An evolutionary history leading to the gene tree of Figure 1.** This history is consistent with the species tree *S* of Figure 1.

Finally, a *simple-event*, will refer to an event acting on a single gene. For example, a simple-deletion will refer to the deletion of a single gene. A simple-deletion event is also referred to as a *loss event.* Moreover, a *general-duplication* will refer to a duplication that does not necessarily place the duplicated genes next to the original copies (not necessarily in tandem). An example of an evolutionary history is given in Figure 2.

We are now ready to formulate our optimization problem:

Minimum-Evolution Problem:

**Input:** An ordered gene tree  and a species tree *S.*

**Output:** A most parsimonious evolutionary history  for  consistent with *S.*

The “most parsimonious” constraint given above can be most naturally expressed in terms of number of events. Alternatively, a cost can be associated to each event depending on the type and size of the event (*i.e.* number of genes affected by this event), and the “most parsimonious” history would be the history of minimum cost, where the cost of a history is simply the sum of costs associated with its events. This latter approach is the one considered in [1].

### The DILTAG method

The DILTAG algorithm [1] allows the inference of a set of most parsimonious histories of duplications, inverted-duplications, inversions and deletions (*i.e.* all events introduced in Definition 1 except speciation), originally acting on a single ancestral gene to produce a given extant TAG cluster represented by a given ordered gene tree (*T*, *O*)*.*

DILTAG proceeds by exploring a “history graph” (search space), where vertices correspond to ordered gene trees and edges correspond to evolutionary events. More precisely, an edge from (*T^i^*, *O^i^*) to (*T^j^*, *O^j^*) is defined if and only if (*T^i^*, *O^i^*) can be transformed into (*T^j^*, *O^j^*) through one event, and each edge is weighted by the cost of its corresponding event. This graph is actually simplified into a finite graph, without loss of information, by considering deletions only in combination with duplication events. The history graph is constructed backwards, *i.e.* starting at vertex (*T*, *O*), and constructing edges in their opposite direction (backward-edges) by exploring the neighbourhood of each vertex.

It is shown in [1] that, given a vertex representing an ordered gene tree (*T*, *O*), its *duplication* and *inverted-duplication* neighbourhoods are both linear (in the size of *T*) in space, where as its *inversion*, *duplication-with-deletion* and *inverted-duplication-with-deletion* neighbourhoods are all quadratic in space. However the size of the whole search-space is clearly exponential, which makes an exhaustive search through the whole graph impossible for gene trees of reasonable size. A greedy heuristic is therefore developed that only conserves, in a queue, the most promising partial evolutionary histories obtained after exploring a given depth of the history-graph.

The input of DILTAG is an ordered gene tree (*T*, *O*) with *n* leaves, and the output is a set of shortest backward-paths in the history graph from (*T*, *O*) to a tree containing a single vertex. For the purpose of our new Multi-DILTAG algorithm, it is easy to modify DILTAG in order to reach an ancestral genome with *g* genes, for any 1 ≤ *g* ≤ *n*: simply stop the procedure as soon as we attain the right number of genes. Notice that the attained ancestor is ordered, *i.e.* defined by an ordered sequence of *g* genes. It can be seen as an ordered tree (*T*′, *O*′) with *T*′ being reduced to a set of *g* vertices and no edges. We will make no distinction between an ordered tree with no edges and a gene order.

In Section 2.5, the input and output of DILTAG will be as follows:

Input: An ordered gene tree (*T*, *O*) and a number *g* of ancestral genes;

Output: The cost of a shortest backward-path from (*T*, *O*) to an ancestral genome with *g* genes, together with the *solution graph* composed by the actual set of shortest paths, and the *solution set* of ancestral gene orders attained.

Finally, we need the following definition for the subsequent developments: given two vertices *x* and *y* of the oriented history graph, if there is an edge oriented from *x* to *y* (there is an evolutionary event transforming *x* into *y*), then we say that *y* is a *predecessor* of *x.*

### A two step method for multiple species

**Figure 3:**
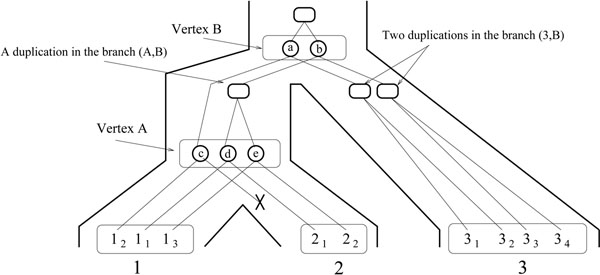
**A reconciliation *R* between the gene tree and the species tree of Figure 1.** Ovals are speciation vertices and squares are duplication vertices. The crossed line indicates a gene loss. The genome sets of internal nodes are *G*(*A*) = {c, *d*, e} and *G*(*B*) = {*a*, *b*}*.* As for the pre-speciation sets, we have *PG*(*A*) = *G*(*B*) = {*a*, *b*}, *PG*(1) = *G*(*A*) = {*c*, *d*, e}, *PG*(3) = *G*(*B*) = {*a*, *b*}, but *PG*(2) = {*d*, *e*}, which is different from *G*(*A*) due to the loss of gene *c*.

Back to our evolutionary model on multiple species, we aim to find a most parsimonious evolutionary history for  that is consistent with *S.* This problem has been considered in [20], but in the more restricted case of *simple-duplications*, and no *inverted-duplications.* A two step methodology has been considered:

1. Reconciliation Step: Ignoring gene orders, infer a history of *simple-general-duplication*, *simple-deletion and speciation* for *T* consistent with *S*, by using a reconciliation approach [30]. Conceptually, a *reconciliation R* between a gene tree *T* and a species tree *S* is a tree accounting for the evolutionary history of the species and all genes of the gene family, including lost and missing gene copies, by simple-general-duplication, speciation and loss. *R* can be “embedded” into *S*, reflecting the duplication and deletion events leading to the observed tree *T.* Such embedding allows to infer the number of genes at the speciation nodes of *S*, as well as the evolutionary relationships between ancestral gene copies. A reconciliation between the gene tree *T* and the species tree *S* of Figure 1 is given in Figure 3. Notice that this reconciliation does not lead to the observed gene order.

2. Minimization Step: Reinserting the gene order and sign information  on the leaves of *S*, infer the order and sign of genes at internal nodes of *S* allowing to minimize the total number of events involved in a history of .

We use the same two-step methodology here. As for the first step, any existing reconciliation method can be used. In particular, the so called Lowest Common Ancestor (LCA) mapping between a gene tree and a species tree, formulated in [31,32] and widely used [32-41], defines a reconciliation tree *R* that minimizes both the simple-general-duplication and simple-deletion events.

In the following developments, we will consider the “embedded” representation of a reconciliation tree *R* into the species tree *S.* More precisely:

• A *leaf* of *R* is an extant gene and maps to a leaf of *S*, *i.e.* the extant genome to which it belongs.

• A *duplication vertex* of *R* is an internal vertex which corresponds to a duplication event. It maps to a branch of *S*, *i.e.* the lineage in which the duplication occurred (see Figure 3).

• A *speciation vertex* of *R* is an internal vertex which corresponds to an ancestral gene at the time of a speciation event. It maps to an internal vertex of *S*, *i.e.* the ancestral genome to which it belongs. It has either one child (in the case of a gene loss), or two children each belonging to a different lineage. The set of speciation vertices mapping to a vertex *A* of *S* is the *genome set G*(*A*) *of A.* If *A* is not the root, let *B* be the father of *A.* Then the *pre-speciation genome set PG*(*A*) *of A* is the subset of *G*(*B*) containing the vertices of *G*(*B*) with a child in the branch (*A*, *B*), in other words, the genes in *G*(*B*) that have not been lost after speciation on the branch going to *A.* We have |*PG*(*A*)| ≤ |*G*(*B*)*|* (see Figure 3).

Considering now the Minimization Step, if only *simple-duplications* are allowed, the problem has been shown in [20] to be equivalent to the one of finding gene orders at internal nodes of *S* minimizing a global inversion distance. In this context, the evolutionary model can be reduced to the one where all duplications occur first, followed by all inversions. The problem is then to find the minimum number of inversions, yielding a forest of simple-duplication trees. Using properties of simple-duplication trees, it is possible to define an exact and efficient algorithm for this problem. All these simplifications and shortcuts do not hold anymore for simultaneous duplications and deletions of multiple genes. In the following section, we focus on the Minimization Step.

### Multi-DILTAG: Extension of DILTAG to multiple species

**Figure 4:**
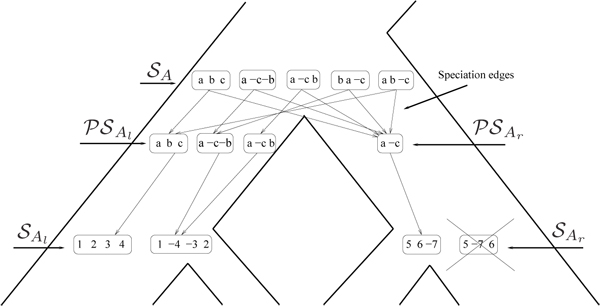
**Computation of the solution set  at the internal node *A* of a species tree *S* by Multi-DILTAG.** DILTAG is executed on each of the two branches (*A_l_*, *A*) and (*A_r_*, *A*), leading to the two pre-speciation sets  and . In each branch, only minimum-cost paths are kept, which explains the removal of one gene order (indicated by a cross) from . The gene missing from the gene order of  is reinserted in all possible positions (and all possible signs, which is not shown), and the resulting set is added to  to form . Appropriate speciation edges are then added from the elements of  to the elements of .

Our algorithm is a generalization of DILTAG that proceeds with the whole species tree *S* and produces a solution set for each internal vertex, and a solution graph with additional speciation edges. Figure 4 illustrates the algorithm execution at each internal vertex *A* of *S.*

Initially, the solution set of each leaf is reduced to the gene order observed at that leaf, and the solution graph is reduced to the set of vertices defined by the ordered gene trees at the leaves. We then extend the solution graph by exploring *S* bottom-up, and for each internal vertex *A*, we compute a solution set  by performing DILTAG respectively on the left branch (*A_l_*, *A*) and right branch (*A_r_*, *A*) of *S* (with *A_l_* and *A_r_* being respectively the left and right child of *A*), and taking, as potential orders at *A*, the union of genome sets *PG*(*A_l_*) and *PG*(*A_r_*) obtained respectively in the left and right branch. However, due to gene losses, gene orders in *PG*(*A_l_*) do not necessarily have the same number of genes as gene orders in *PG*(*A_r_*)*.* We therefore consider all possible extensions of gene orders, by reinserting lost copies in any possible way, and take the union of all sets obtained as the solution set . We then define a single “speciation edge” in the solution graph from each vertex representing a gene order in  to each vertex representing a gene order in *PG*(*A_l_*) ∪ *PG*(*A_r_*)*.* As the only evolutionary events likely to have occurred on these edges of the history graph are inversions and deletions, we label each speciation edge (*x*, *y*) by the minimum Inversions+Deletions (ID) distance allowing to transform *x* into *y.* In the literature, the problem of computing the ID-distance between two permutations has already been considered, and a polynomial-time algorithm exists [42,43].

More precisely, the Multi-DILTAG algorithm traverses the tree bottom-up, and for each internal node *A* proceeds as follows:

1. For each of *s* ∈ {*l*, *r*}, execute DILTAG on each element of , and stop as soon as the attained gene order contains |*PG*(*A_s_*)| genes. The set of all ancestral gene orders obtained (output of DILTAG) form an initial *pre-speciation* set , further truncated as follows: if *MIN* is the minimum cost obtained over all elements of , we remove from  all elements *O* that are not attained with the cost *MIN.* Moreover, we remove from the partial current solution graph all the predecessors of *O* that are not linked to another element of  by a minimum-cost path.

2. For each of *s* ∈ {*l*, *r*}, construct the set  by replacing each gene order *O* of  by the set of all possible orders obtained from *O* by inserting the genes lost on the branch (*A*, *A_s_*)*.*

3. Compute . The solution graph is extended by adding one vertex per each element of .

4. Let , and suppose, w.l.o.g. that . Then complete the solution graph by constructing an oriented “speciation edge” from *O* to the vertex corresponding to its originating order in *A_l_*, and an oriented edge from *O* to the vertex corresponding to each element of  giving rise to the minimum ID-distance with *O.*

## Results and discussion

We implemented our algorithm and applied it to simulated data sets to evaluate its execution time and precision in terms of the number and size distribution of the inferred duplications. Then, we applied it to the protocadherin gene clusters of four mammalian species to infer the duplication size distribution and the number of events that occurred in the evolutionary history of these species.

### Experiments on simulated data sets

Ordered gene trees were generated by simulating evolutionary histories consistent with balanced species trees of 2, 4 or 8 leaves. Note that we also tested our algorithm on unbalanced species trees to ensure that it does not affect its accuracy (data not shown). Unless stated otherwise, the size of each event was sampled according to a geometric distribution of parameter *p* = 0.5, truncated by the number of genes in the ancestral cluster immediately preceding this event. The geometric distribution was chosen to represent biological data, in which smaller events are observed more frequently. We also tested *p* = 0.3 and *p* = 0.8, which give respectively more and less large events, and the results were similar (data not shown). All the results shown below are averaged over 50 replicates.

Similarly to the DILTAG algorithm, we define the penalty cost of an event *e* of size *m* (acting on a segment of *m* genes) as *α_e_* + *mβ_e_*, where *α_e_* is the opening cost and *β_e_* the extension cost of *e*. Our results were obtained with the same values used in [1] to test the DILTAG algorithm, namely:

• *α_t–dup_* = 100; *β_t–dup_* = 1,

• *α_i–dup_* = 100; *β_i–dup_* = 1,

• *α_del_* = 500; *β_del_* = 1,

• *α_inv_* = 500; *β_inv_*= 1.

#### Execution time

**Figure 5:**
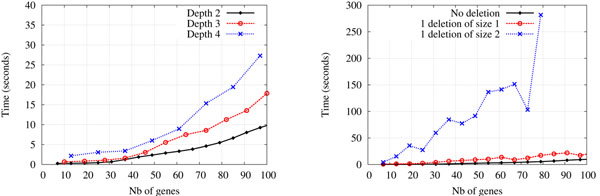
**Execution time.** Left: Execution time of Multi-DILTAG on genomes containing a fixed number of genes on all the leaf genomes. Balanced species trees of maximum depth 2 (2 leaves), 3 (4 leaves) and 4 (8 leaves) were generated. Right: Execution time of Multi-DILTAG on species trees with two leaves, for simulated histories with no deletion (the same curve as the one indicated by a plain black line on the left diagram), 1 deletion of size 1 and 1 deletion of size 2.

Our algorithm was implemented in C++ and runs on a typical Linux workstation. Figure 5 shows the execution time of Multi-DILTAG. The left diagram shows results for balanced species trees of 2, 4 and 8 leaves. The depth *d* of the extant genomes for trees with 2, 4 and 8 leaves are respectively 2, 3 and 4. We generated histories with *n* single, *n* double tandem duplications (simultaneous duplication of 2 genes) and 2 inversions on each branch of the species tree. At each step in the curves, *n* is incremented by 1 and thus the number of genes in each extant genome is equal to 3*dn* + 1. Note that this is the only experiment in which we used fixed tandem duplication sizes (1 or 2), and we did this only to get the same number of genes in every genome.

Figure 5 right then shows the effect of introducing deletions. Only histories with 2 extant genomes were generated, and we plotted the running times for simulated histories containing no deletion, 1 deletion of size 1 and 1 deletion of size 2.

Clearly the execution time of Multi-DILTAG is exponential in the number of genes in extant genomes. Nevertheless, it is possible to get results in under 30 seconds for a family of approximately 100 genes in 8 species. On the other hand, deletions of size greater than 1 slows down Multi-DILTAG dramatically. The idea of considering all possible extensions of gene orders, by reinserting lost copies in any possible way, results in an exponential number of orders in the number of copies to reinsert and the size of the orders in which we make the insertions.

#### Number of duplications

**Figure 6:**
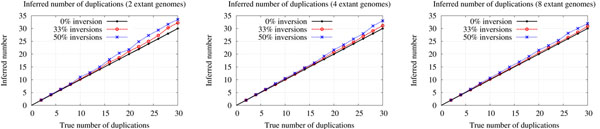
**Number of duplications.** Inferred number of duplications (direct + inverted) for histories containing duplications and respectively 0 %, 33 % and 50 % of inversions. Left: Two extant genomes. Center: Four extant genomes. Right: Eight extant genomes.

We now evaluate the ability of Multi-DILTAG to infer the correct total number of duplications (direct + inverted). We simulated evolutionary histories containing as many duplications as inverted duplications with 2 (Figure 6 left), 4 (Figure 6 center) and 8 (Figure 6 right) extant genomes, and we plotted the total number of duplications inferred for histories generated with 0 %, 33 % and 50 % of inversions.

More precisely, for each *x*, we generate a history with a total of *x* duplications together with 0, *x*/2 or *x* inversions, respectively leading to the curves for 0 %, 33 % and 50 % of inversions. The total number of events performed for each value of *x* is distributed evenly on the branches of the species tree.

As we see, Multi-DILTAG is almost perfect in inferring the total number of duplications when there are no inversions. The presence of inversions induces a small overestimation in the inferred number of duplications. As noticed in [1], this can be explained by the size limit of the DILTAG priority queue used to explore the search space and the chosen cost configuration, which may lead to choosing a history with more duplications in order to infer fewer inversions.

Notice that the overestimation is a little bit more pronounced in Figure 6 left. This can be easily explained by the fact that there are fewer branches in the balanced species tree containing 2 extant genomes than in the ones of 4 and 8 extant genomes. Therefore, for the same total number of duplications, more inversions are present on each branch of the smallest species tree.

#### Duplication size distribution

**Figure 7:**
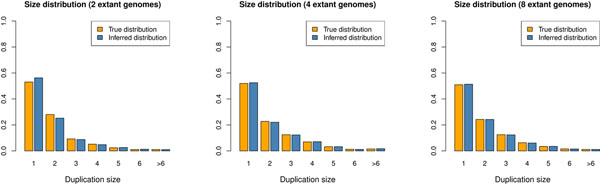
**Comparison between the true and the inferred duplication size distribution.** Histories were generated with 4 tandem duplications, 1 inverted duplication, 1 inversion and 1 deletion of size 1 or 2 on each branch of the species tree. Left: Two extant genomes. Center: Four extant genomes. Right: Eight extant genomes.

Finally, we measure the accuracy of Multi-DILTAG for inferring the duplication size distribution. Histories containing 2 (Figure 7 left), 4 (Figure 7 center) and 8 (Figure 7 right) extant genomes were generated. In all cases, 4 tandem duplications, 1 inverted duplication, 1 inversion and 1 deletion of size 1 or 2 were simulated on each branch of the corresponding balanced species tree.

Clearly, Multi-DILTAG is able to infer the duplication size distribution very accurately for the three data sets. We can only observe a slight overestimation of duplications of size 1 and underestimation of duplications of size 2.

We do not report the correctness of the inferred duplication events because a lot of equivalent optimal evolutionary histories are obtained by Multi-DILTAG, so it is possible that most of the inferred duplications do not correspond to the simulated duplications.

### Experiments on the protocadherin gene clusters

We applied Multi-DILTAG to the three protocadherin (Pcdh) gene clusters (*α*, *β* and *γ*) in human, chimpanzee, mouse and rat (for the *α* cluster only). It is believed that protocadherins play a role in synaptic development and neuronal survival [44-46]. Each gene in the protocadherin clusters consists of a single *variable* exon. In the *α* and *γ* clusters only, there are three additional *constant* exons at their 3’ end that are alternatively cis-spliced to each variable exon. This kind of genomic organization suggests a mode of evolution through tandem duplications and deletions of the variable exons in each cluster (inversions and inverted duplications are not allowed here as they would be deleterious).

We downloaded most of the protein sequences for the three protocadherin gene clusters from the UCSC Genome Browser (http://genome.ucsc.edu/) for human (February 2009, hg19), chimpanzee (October 2010, panTro3), mouse (July 2007, mm9) and rat (November 2004, rn4). Missing genes in the downloaded sequences for chimpanzee were downloaded manually from UniProt (http://www.uniprot.org/). The rat *β* and *γ* clusters were discarded from our experiments because some gene sequences could not be found. We restricted our analysis to the regions of the variable exons encoding ectodomains 2 and 3, since it has been shown that these regions are the most divergent and retain most of the phylogenetic signal [28,47]. The human and mouse CDH12 genes were used as an outgroup. The protein sequences were aligned with ProbCons version 1.12 [48] and rooted gene trees were obtained with MrBayes version 3.1.2 [49], using the Jones-Taylor-Thornton substitution matrix [50] and 500,000 MCMC iterations.

We then applied Multi-DILTAG to the first hundred most probable trees obtained for each Pcdh cluster, averaging our results proportionally to the posterior probability of each tree. However, recall that our algorithm computes the minimal ID-distance on each speciation edge of the solution graph. As mentioned earlier, inversions are not allowed in the case of the protocadherin gene clusters, so the inferred evolutionary histories that contain inversions are discarded from our results. The presence of these inversions might be the result of an incorrect input gene tree, or might simply show that Multi-DILTAG is unable to find the correct evolutionary history for this input tree. Note that only 14 gene trees (on a total of 300) caused inversions to appear in the inferred histories. The posterior cumulative probability (according to MrBayes) of the considered gene trees for the *α*, *β* and *γ* clusters are respectively 0.504, 0.690 and 0.409.

To ensure that the results do not significantly depend on the choice of the cost parameters, we used three different configurations: (*α_del_* = 500 ; *β_del_* = 1), (*α_del_* = 250 ; *β_del_* = 250) and (*α_del_* = 1 ; *β_del_* = 500).

**Figure 8:**
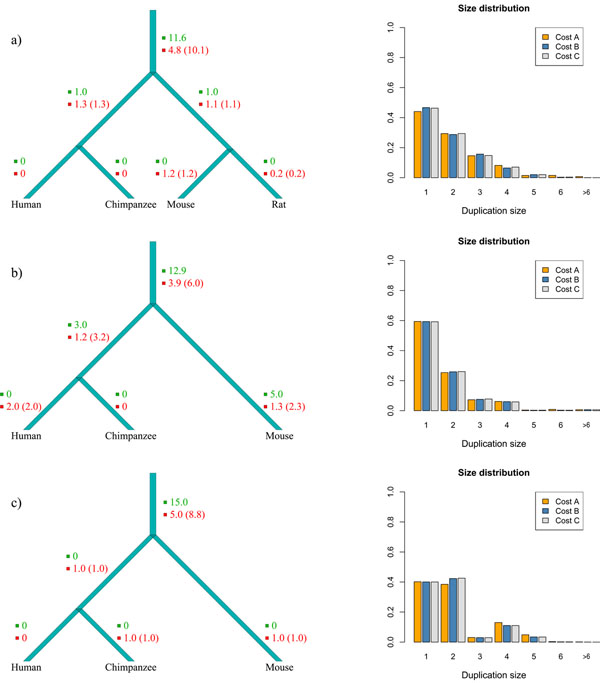
**Results for the Pcdh-*α* (a), Pcdh-*β* (b) and Pcdh-*γ* (c) clusters.** Left: Estimated number of events on each branch of the species tree for cost configuration A (*α_del_* = 500 ; *β_del_* = 1). The number of tandem duplications is written in green, the number of deletions is written in red and the number of gene losses is shown in parentheses. Right: Inferred duplication size distributions for the three different cost configurations considered: A (*α_del_* = 500 ; *β_del_* = 1), B (*α_del_* = 250 ; *β_del_* = 250) and C (*α_del_* = 1 ; *β_del_* = 500).

The number of events inferred by Multi-DILTAG on each branch of the species tree and the duplication size distributions for the three protocadherin gene clusters are presented in Figure 8.

As we could expect from the well-conserved number of genes between the studied species, almost all the events occurred on the branch above the last common ancestor of these species (Figure 8 left). We can also see that there is an important fraction of multiple gene duplications in the size distributions (Figure 8 right). Another interesting fact is that approximately the same number of double tandem duplications and single tandem duplications were inferred in the Pcdh-*γ* cluster (Figure 8 (c) right). This tends to confirm the hypothesis suggested in [51] that the Pcdh-*γ* cluster evolved by duplications involving pairs of genes.

## Conclusions

We presented Multi-DILTAG, a generalization of DILTAG for the study of the evolutionary history of a set of orthologous TAG clusters in multiple species, with an evolutionary model allowing for simple or multiple tandem duplications, direct or inverted, simple or multiple deletions, and inversion events. Our results showed that our algorithm is very robust in inferring the number and size distribution of duplications. We then applied Multi-DILTAG to the protocadherin gene clusters of human, chimpanzee, mouse and rat to estimate the number of events among the different branches of the species tree and the duplication sizes. A short-term future work will concern the application of our algorithm to other sets of orthologous gene clusters.

However, a clear limitation of Multi-DILTAG is the time complexity of the approach taken to deal with deleted genes. An important future work will be to develop a fast heuristic to find an optimal set of extensions of gene orders without reinserting the lost copies in any possible way.

## Competing interests

The authors declare that they have no competing interests.
